# Serologic Screening of Severe Acute Respiratory Syndrome Coronavirus 2 Infection in Cats and Dogs during First Coronavirus Disease Wave, the Netherlands

**DOI:** 10.3201/eid2705.204055

**Published:** 2021-05

**Authors:** Shan Zhao, Nancy Schuurman, Wentao Li, Chunyan Wang, Lidwien A.M. Smit, Els M. Broens, Jaap A. Wagenaar, Frank J.M. van Kuppeveld, Berend-Jan Bosch, Herman Egberink

**Affiliations:** Utrecht University, Utrecht, the Netherlands

**Keywords:** severe acute respiratory syndrome coronavirus 2, SARS-CoV-2, coronaviruses, viruses, coronavirus disease, COVID-19, respiratory infections, cats, dogs, serologic screening, ELISA, virus neutralization, receptor-binding domain, nucleocapsid protein, zoonoses, the Netherlands

## Abstract

Severe acute respiratory syndrome coronavirus 2 (SARS-CoV-2) can infect many animal species, including minks, cats, and dogs. To gain insights into SARS-CoV-2 infections in cats and dogs, we developed and validated a set of serologic assays, including ELISA and virus neutralization. Evaluation of samples from animals before they acquired coronavirus disease and samples from cats roaming SARS-CoV-2–positive mink farms confirmed the suitability of these assays for specific antibody detection. Furthermore, our findings exclude SARS-CoV-2 nucleocapsid protein as an antigen for serologic screening of cat and dog samples. We analyzed 500 serum samples from domestic cats and dogs in the Netherlands during April–May 2020. We showed 0.4% of cats and 0.2% of dogs were seropositive. Although seroprevalence in cats and dogs that had unknown SARS-CoV-2 exposure was low during the first coronavirus disease wave, our data stress the need for development of continuous serosurveillance for SARS-CoV-2 in these 2 animal species.

A novel human coronavirus (HCoV), severe acute respiratory syndrome coronavirus 2 (SARS-CoV-2), emerged in Wuhan, China, during December 2019 and caused a severe pandemic of coronavirus disease (COVID-19) (*1*,*2*). As of January 2021, SARS-CoV-2 had spread to 223 countries and caused >88 million infections, which occurred by human-to-human transmission and mostly affected elderly and immunocompromised persons (*3*).

SARS-CoV-2 is a zoonotic virus and was shown able to infect many animal species, such as cats, dogs, ferrets, fruit bats, hamsters, and several nonhuman primates under experimental condition (*4*–*6*). Recently, transmission of SARS-CoV-2 from humans to cats and dogs shown by viral RNA or antibody detection has been reported, resulting in asymptomatic infections in dogs, and symptomatic and asymptomatic infections in cats (*7*–*15*). There is currently no evidence that pets play a role in spread of the virus. Nevertheless, close contacts between owners and pets and interactions between dogs and cats from different households raise the question about the role of these animals in SARS-CoV-2 transmission.

Diagnosis of SARS-CoV-2 is currently made by using molecular assays, such as real-time PCR. However, viral nucleic acid is only detectable within a limited timeframe after infection, and serologic screening of SARS-CoV-2–specific antibodies in cats and dogs is needed for insights into the prevalence of this infection and possible modes of transmission (human-to-animal, animal-to-animal, and animal-to-human).

We developed and validated SARS-CoV-2–specific serologic assays. Serum samples were first tested with ELISAs by using different antigens, including spike protein subunit (S1) of endemic feline and canine coronaviruses and SARS-CoV-2 antigens (S1, receptor binding domain [RBD], and nucleocapsid [N] protein), and subsequently analyzed by using virus neutralization titer (VN) assays with SARS-CoV-2 spike pseudotyped virus. Using these assay platforms, we conducted serosurveillance study of SARS-CoV-2 in cats and dogs of unknown SARS-CoV-2 exposure during the first wave of COVID-19 pandemic (April–May 2020) in the Netherlands.

## Materials and Methods

### Serum Samples

Cat and dog serum samples collected during 2019 (pre–COVID-19 cohort, n = 45 each) were obtained from the serum bank of Utrecht University (Utrecht, the Netherlands). Paired and postinfection serum samples of feline coronavirus (FCoV) type I–infected specific pathogen-free (SPF) cats (n = 9) were obtained from SPF cats infected with FCoV strain UU2 or RM in a previous study (*16*). The SARS-CoV-2–exposed cohort consisted of 44 serum samples from stray cats roaming on SARS-CoV-2–positive mink farms (*17*) and 1 serum sample of a dog from a COVID-19–confirmed household. The 2020 cohort is composed of domestic cat and dog serum or plasma samples (n = 500 each) that were sent to the University Veterinary Diagnostic Laboratory or the Veterinary Microbiological Diagnostic Center at Utrecht University for routine diagnostics during April–May 2020. Data on SARS-CoV-2 exposure of these animals was not available. All samples were stored at −20°C until use and heat-inactivated at 56°C for 30 min before use.

### Antigen Preparation

We produced streptavidin–tagged SARS-CoV-2 S1 and RBD proteins in eukaryotic cells as described (*18*,*19*), and cloned and similarly produced streptavidin-tagged bovine coronavirus (BCoV) S1 and HCoV-229E S1. SARS-CoV-2 N protein was obtained from Sino Biological (https://www.sinobiological.com). We produced mouse Fc-tagged FCoV type I S1, FCoV type II S1, or BCoV S1 proteins as described (*20*). Vesicular stomatitis virus (VSV) pseudotyped with SARS-CoV-2 S protein (SARS2-VSV) was prepared as described (*18*) and titrated on Vero E6 cells.

### ELISA

We first screened samples from the 3 cohorts with indirect ELISAs for the different proteins as described (*20*). In brief, high-binding microtiter plates were coated with equal molar amounts of protein (1 pmol/ well after optimizing by using checkerboard titration), diluted in phosphate-buffered saline, and blocked with blocking buffer (phosphate-buffered saline containing 0.05% Tween-20 and 5% milk powder). A standard 1:50 dilution of serum samples or serial 2-fold dilutions of serum samples starting at a 1:50 dilution were added to the wells. After incubation for 1 h at 37°C, plates were washed and subsequently incubated with horseradish peroxidase (HRP)–conjugated secondary antibody (1:4,000 for goat anti-cat IgG/HRP; Rockland Immunochemicals, Inc., https://rockland-inc.com) and 1:6,000 for goat anti-dog IgG/HRP; Cappel, http://ziobio.com) diluted in blocking buffer for 1 h at 37°C. Peroxidase reactions were visualized by incubation with 3,3′,5,5′-tetramethylbenzidine (10 min at room temperature) and quenching with sulfuric acid. Optical densities (ODs) were measured at 450 nm. Cutoff values were determined at 6-fold SDs above the mean value of reactivity of all negative serum samples from the pre–COVID-19 cohort (*19*).

### S1 Adsorption Assay

To verify that the 2 betacoronavirus infections in dogs (SARS-CoV-2 and canine respiratory coronavirus [CRCoV]) can be distinguished serologically, we designed an antigen S1 adsorption assay. We incubated serum samples with Strep-Tactin Sepharose Beads (IBA Lifesciences, https://www.iba-lifesciences.com) conjugated with S1 protein of SARS-CoV-2, BCoV, or HCoV-229E and titrated mock-absorbed and protein-absorbed serum samples in the ELISA. We expressed IgG titers as the reciprocal of highest serum dilution resulting in OD values above the cutoff value.

### Virus Neutralization Assay

**Figure 6:**
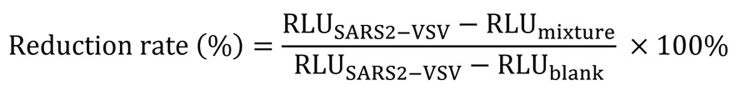
RLU reduction rates of samples were calculated by using this formula.

We conducted a VN assay by using luciferase-encoding VSV particles pseudotyped with S protein of SARS-CoV-2 (SARS2-VSV), which was conducted on Vero E6 cells in a 96-well plate (*18*). Antigenicity of SARS2-VSV was validated previously, and VN titers (VNTs) for SARS2-VSV correlated well with those for live SARS-CoV-2 (*18*). Samples (starting at a 1:8 dilution) were serial diluted 2-fold and mixed 1:1 with SARS-2-VSV. Mixtures were preincubated at 37°C for 1 h and used for inoculation on cells. Twenty-four hours postinfection, cells were lysed and relative luminescence units (RLU) of luciferase activity was determined as described (*18*). RLU reduction rates of samples were calculated by using the formula (Figure 6)

Sample neutralization titers were determined by using the reciprocal of the highest dilution that resulted in >50% reduction of luciferase activity. A VNT >16 was considered positive (*21*).

### Statistical Analyses

All statistical analyses were performed by using Prism version 7.04 for Windows (GraphPad, https://www.graphpad.com). The Pearson correlation coefficient was calculated to determine the correlation between different ELISA ODs and VNTs. The 95% CIs were determined by using the modified Wald method.

## Results

### Pre–COVID-19 Cohort

**Figure 1:**
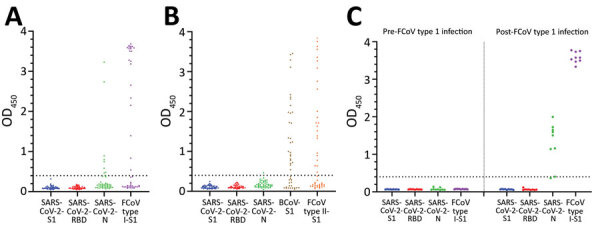
ELISA reactivities against different antigens of pre–coronavirus disease (COVID-19) cat and dog serum samples and paired samples of FCoV type I infection, the Netherlands. A) Reactivities of pre–COVID-19 cat serum samples against SARS-CoV-2 S1, RBD, N, and FCoV type I S1. B) Reactivities of pre–COVID-19 dog serum samples against SARS-CoV-2 S1, RBD, N, BCoV S1, and FCoV type II S1. C) Reactivities of paired SPF cat serum samples (left panel) and FCoV type I–specific serum samples (right panel) to SARS-CoV-2 S1, subunit; RBD, N, and FCoV S1 protein levels were determined by ELISA. Dotted lines indicate positive cutoff levels. BCoV, bovine coronavirus; FCoV, feline coronavirus; N, nucleocapsid; OD, optical density; RBD, receptor-binding domain; S1, spike protein subunit 1; SARS-CoV-2, severe acute respiratory syndrome coronavirus 2; SPF, specific pathogen free.

Serum samples from the pre–COVID-19 cohort were tested against SARS-CoV-2 antigens to screen for potential cross-reactive antibodies elicited by endemic coronaviruses in cats and dogs because they are natural reservoirs of several coronaviruses (i.e., FCoV [genus *Alphacoronavirus*] in cats, canine coronavirus [CCoV; genus *Alphacoronavirus*] and CRCoV [genus *Betacoronavirus*] in dogs) (*20*,*22*,*23*). We summarized sequence identities of SARS-CoV-2 antigens used and matching endemic coronavirus antigens (Table 1). FCoV type I S1 was used as an additional antigen to assess the reactivity of cat serum samples. For dog serologic analysis, FCoV type II S1 (92.1% similar to S1 of CCoV) was used as a proxy antigen for CCoV, and BCoV S1 (95.7% similar to S1 of CRCoV) was used as a proxy antigen for CRCoV. Many serum samples were positive for FCoV and BCoV S1, but all samples were negative for antibodies against SARS-CoV-2 S1 and RBD (Figure 1). Because of limited sample volumes, a selection of serum samples (n = 34 for cats and n = 24 for dogs) was tested for SARS-CoV-2 S–bearing VSV pseudovirus (SARS2-VSV) neutralization, and all showed negative results (VNT <16).

**Table 1:** Percentage amino acid identity of canine and feline coronavirus spike and nucleocapsid proteins with SARS-CoV-2 proteins, the Netherlands*

Genus	Virus	SARS-CoV-2			
		N	S	S1	RBD
Betacoronavirus	CRCoV	32.4	28.5	20.0	15.6
Alphacoronavirus	FCoV type I	29.0	24.0	16.8	7.7
Alphacoronavirus	FcoV type II	27.8	25.3	17.7	8.9
Alphacoronavirus	CCoV	28.0	25.1	16.9	8.9

*SARS-CoV-2, CRCoV, FCoV type I, FCoV type II, CCoV (GB: NC_045512.2, JX860640.1, FJ938060.1, AY994055.1, KC175341.1). Amino acid sequences were aligned by using Clustal W (https://www.ebi.ac.uk/Tools/msa/clustalo), and pairwise identities were calculated by using the needle method in the EMBOSS pairwise alignment algorithms program (https://www.ebi.ac.uk/Tools/psa/emboss_needle). CCoV, canine coronavirus; CRCoV, canine respiratory coronavirus; FCoV, feline coronavirus, N, nucleocapsid protein; RBD, receptor-binding domain; S, spike protein; SARS-CoV-2, severe acute respiratory syndrome coronavirus 2; S1, spike protein subunit 1.

A total of 8 (17.8%) of 45 pre–COVID-19 cat serum samples and 1 (2.2%) of 45 dog serum samples showed positive results in the SARS-CoV-2 N protein ELISA (Figure 1, panels A, B). To explore this finding, we analyzed paired serum samples of SPF cats infected with FCoV (Figure 1, panel C). Serum samples from uninfected SPF cats were negative. After FCoV infection, 8 (88.9%) of 9 cats had antibodies reacting with SARS-CoV-2 N protein. When compared with S1 and RBD proteins, we found that the N protein was more conserved among CoVs (Table 1), which might explain the cross-reactivity between FCoV and SARS-CoV-2 detected in our ELISAs.

### SARS-CoV-2–Exposed Cohort

**Figure 2:**
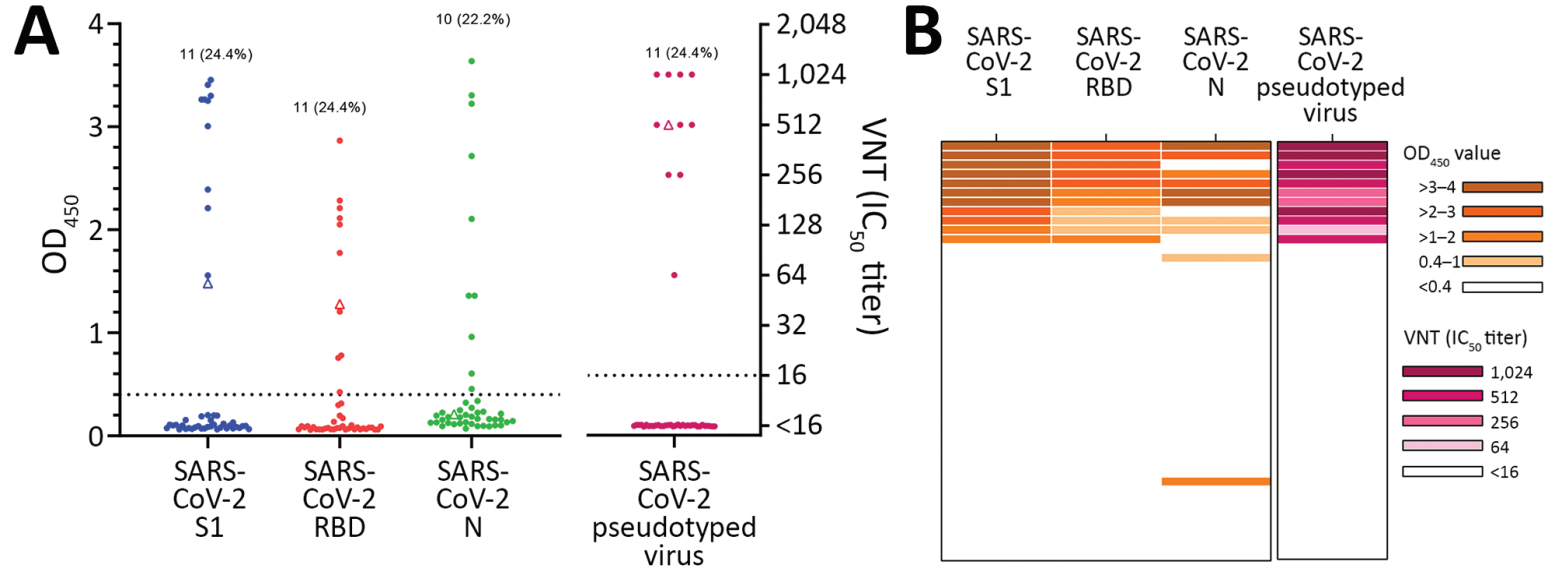
Serologic analyses of cat and dog serum samples from SARS-CoV-2–exposed cohort, the Netherlands. A) ELISA against SARS-CoV-2 S1, RBD, and N proteins, and VN analysis with SARS-CoV-2 pseudotyped virus. Dots indicate cat serum samples (n = 44) and triangle indicates dog sample (n = 1). B) Combination of results tested by different assays expressed as a heatmap. Dotted lines indicate positive cutoff levels. IC_50_, 50% inhibitory concentration; N, nucleocapsid; OD, optical density; RBD, receptor-binding domain; S1, spike protein subunit 1; SARS-CoV-2, severe acute respiratory syndrome coronavirus 2; VN, virus neutralization.

We tested the serum of a dog from a COVID-19–confirmed household, as well as serum samples from SARS-CoV-2–exposed stray cats found in the surroundings of SARS-CoV-2–positive mink farms (*17*). These cats had access to the stables and cages in which the minks were housed. This cohort was expected to contain a higher number of SARS-CoV-2–positive samples because of close contact between the cats and minks and the dog and its owner and was a source of suitable samples for validation of our ELISA and VNT. A total of 11 (24.4%, 95% CI 14.1%–38.8%) of 45 serum samples from 10 cats and 1 dog were positive by ELISA for SARS-CoV-2 S1 and RBD, and 10 (22.2%, 95% CI 12.4%–36.5%) of 45 samples (were reactive against SARS-CoV-2 N protein (Figure 2, panel A). All S1- and RBD-positive samples could neutralize SARS2-VSV infections, but N protein positivity and VN ability were not well associated (Figure 2, panel B).

**Figure 3:**
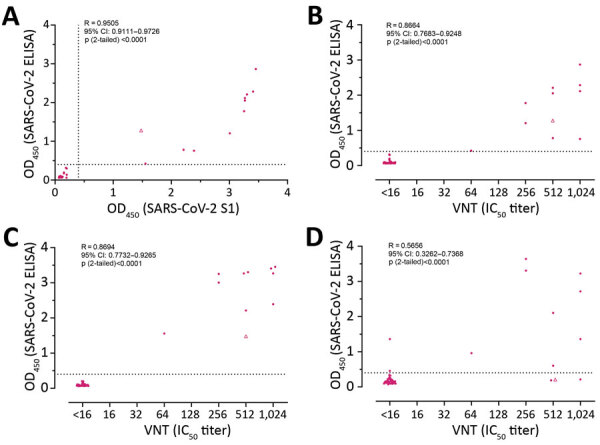
Pairwise correlation analyses of reactivities acquired for serologic analyses of SARS-CoV-2–exposed cohort, the Netherlands. Pearson correlation coefficient was calculated to determine the correlation between the reactivities of RBD ELISA vs. S1 ELISA (A), RBD ELISA vs. VNT (B), S1 ELISA vs. VNT (C), and N ELISA vs. VNT (D). Cat serum samples (n = 44) were indicated in dots and the dog sample (n = 1) in triangle. Dotted lines show the positive cutoff levels. IC_50_, 50% inhibitory concentration; N, nucleocapsid; OD, optical density; RBD, receptor-binding domain; S1, spike protein subunit 1; SARS-CoV-2, severe acute respiratory syndrome coronavirus 2; VNT, virus neutralization titer.

OD values obtained for the SARS-CoV-2 S1 and RBD ELISAs showed a strong correlation with each other (R = 0.95), and both correlated well with VNT (R = 0.87) (Figure 3, panels A–C). Conversely, only a poor correlation was observed between OD values obtained for N protein ELISA and VNT (R = 0.57) (Figure 3, panel D). These data validate SARS-CoV-2 S1 and RBD and exclude N protein as antigen for serologic screening of cat and dog serum samples.

### SARS-CoV-2 Seroprevalence in Domestic Cats

**Figure 4:**
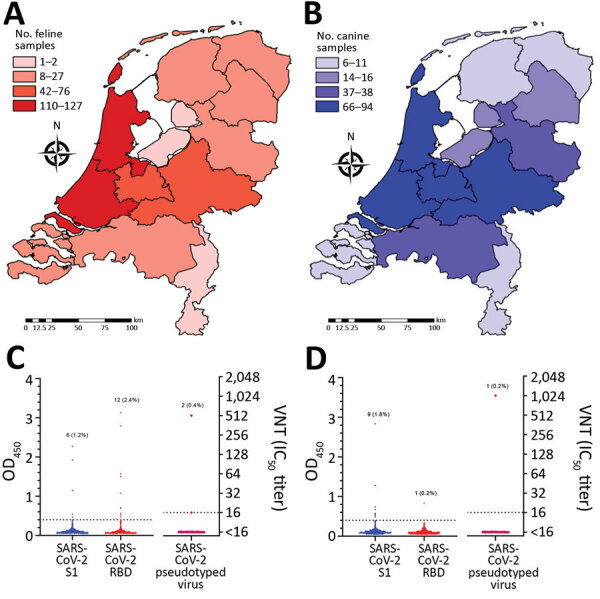
Geographic coverage and serologic analysis of cat (A, C) and dog (B, D) samples of 2020 cohorts for SARS-CoV-2, the Netherlands. A, B) Geographic distribution. Choropleth maps were produced by using ArcGIS version 9.3.1 (Esri, https://www.esri.com). C, D) ELISA and VNT analysis. Number and percentages of positive samples are indicated. Dotted lines indicate positive cutoff levels. Samples that had a VNT >16 were considered positive. IC_50_, 50% inhibitory concentration; OD, optical density; RBD, receptor-binding domain; S1, spike protein subunit 1; SARS-CoV-2, severe acute respiratory syndrome coronavirus 2; VNT, virus neutralization titer.

A total of 500 cat samples from the 2020 cohort were tested by using SARS-CoV-2 S1 and RBD ELISAs (Figure 4, panels A, C). FCoV type I S1 was included as an additional antigen in the ELISA, and 71% of cat samples were FCoV type I antibody positive. Six cat samples were positive for SARS-CoV-2 S1 and RBD, and an additional 6 samples were positive only for RBD (Figure 4, panel C). We have summarized results of different tests (Table 2). We tested by VN assay all samples positive for SARS-CoV-2 S1 or RBD by ELISA , together with 50 randomly chosen samples that showed negative results in the S1 and RBD ELISAs. Two samples that reacted with SARS-CoV-2 S1 and RBD were able to neutralize SARS2-VSV infection, and all ELISA-negative samples were also negative in the VN assay (Table 2; Figure 4, panel C). On the basis of results obtained for SARS-CoV-2–exposed animals, we defined a seropositive sample as any sample being ELISA positive for SARS-CoV-2 S1 and RBD, and with a VNT >16. Samples that did not consistently show diagnostic thresholds (ELISA positive for S1 and RBD, but VNT <16) were considered as being suspected (Table 2). Accordingly, 2 (0.4%, 95% CI 0.01%–1.55%) of 500 domestic cat samples with unknown SARS-CoV-2 exposure had reached the diagnostic thresholds, and henceforth were confirmed as seropositive. Four serum samples were defined as suspected.

**Table 2:** Serologic results for animal samples tested in different serologic assays, the Netherlands*

Animal	Cohort	SARS-CoV-2 S1 ELISA†	SARS-CoV-2 RBD ELISA†	VNT‡	No. samples	Result
Cat	SARS-CoV-2 exposed, n = 44	+	+	+	10	Seropositive
		–	–	–	34	Seronegative
	2020, n = 500	+	+	+	2	Seropositive
		+	+	–	4	Suspected
		–	+	–	6	Seronegative
		–	–	–/NA	488	Seronegative
Dog	SARS-CoV-2 exposed, n = 1	+	+	+	1	Seropositive
	2020, n = 500	+	+	+	1	Seropositive
		+	–	–	8	Seronegative
		–	–	–/NA	491	Seronegative

*NA, not applicable; RBD, receptor-binding domain; SARS-CoV-2, severe acute respiratory syndrome coronavirus 2; S1, spike protein subunit 1; VNT, virus neutralization titer; –, negative; +, positive. †An ELISA optical density value greater than or equal to the cutoff value of 0.4 is a positive result, and an ELISA OD value less than the cutoff value is a negative result.  ‡Neutralization titers of samples were determined by using the reciprocal of the highest dilution that resulted in >50% reduction of luciferase activity in pseudovirus virus neutralization. A VNT greater than or equal to the cutoff value of 16 is a positive result, and a VNT less than the cutoff value is a negative result.

### SARS-CoV-2 Seroprevalence in Domestic Dogs

We tested 500 dog samples by using the SARS-CoV-2 S1 and RBD ELISAs (Figure 4, panels B, D). FCoV type II S1 was included as an additional antigen, and results showed that 40.4% were positive for FCoV type II S1 antibody (indicator of CCoV exposure). Nine samples were positive for SARS-CoV-2 S1, of which only 1 was positive for RBD (Table 2; Figure 4, panel D). Only the sample that reacted with SARS-CoV-2 S1 and RBD was able to neutralize SARS2-VSV. Randomly chosen ELISA negative samples (n = 50) were negative in the VN assay (Table 2; Figure 4, panel D). Thus, 1 (0.2%, 95% CI, <0.01%–1.24%) of 500 of domestic dog samples with unknown SARS-CoV-2 exposure was considered seropositive.

### Confirmation of SARS-CoV-2–Specific Antibodies in Dog Samples by using Adsorption Assays

**Figure 5:**
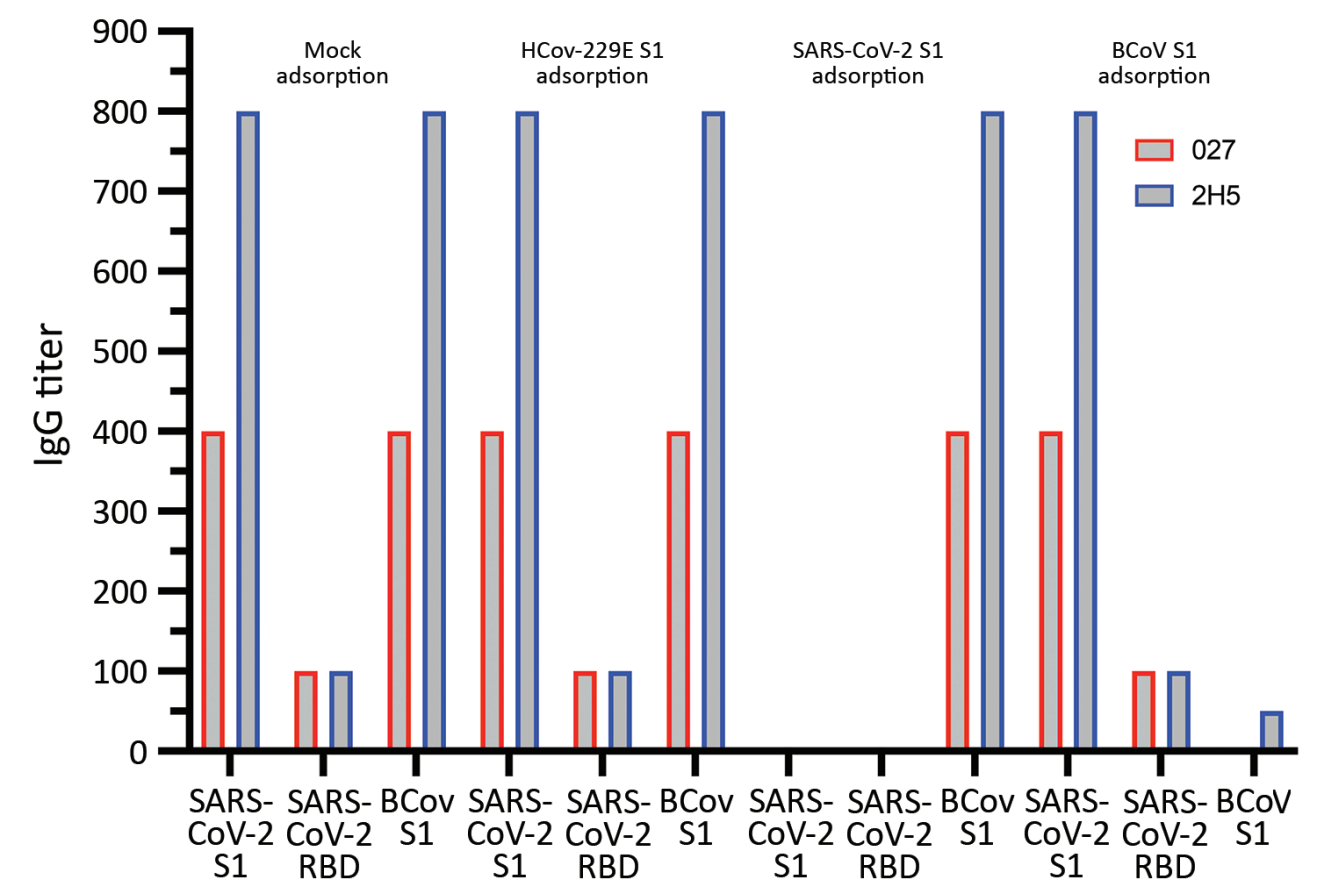
Corroboration of SARS-CoV-2 seropositivity in dog samples with adsorption assays, the Netherlands. ELISA reactivities of the 2 positive dog samples were determined against SARS-CoV-2 S1, RBD, and BCoV S1 after mock adsorption or adsorption with HCoV-229E S1, SARS-CoV-2 S1, or BCoV S1 proteins. The 2 seropositive dog samples (027 and 2H5) are from the SARS-CoV-2–exposed cohort and 2020 cohort, respectively BCoV, bovine coronavirus; HCoV, human coronavirus; RBD, receptor-binding domain; S1, spike protein subunit 1; SARS-CoV-2, severe acute respiratory syndrome coronavirus 2.

The 2 seropositive dog samples also contained antibodies against CRCoV, which belongs to genus *Betacoronavirus*, as does SARS-CoV-2 (Figure 5). To corroborate SARS-CoV-2 seropositivity, we performed an antigen S1 adsorption assay with S1 proteins of SARS-CoV-2 or BCoV (proxy for CRCoV). HCoV-229E (genus *Alphacoronavirus*) S1 was used as a control. Although adsorption of 229E S1 did not change ELISA reactivity for serum samples against SARS-CoV-2 and BCoV antigens, adsorption of SARS-CoV-2 and BCoV S1 specifically removed ELISA reactivity against the corresponding protein (Figure 5). These data confirmed that ELISA reactivity against SARS-CoV-2 for these 2 dog samples is specific, in accordance with the screening of CRCoV-positive pre–COVID-19 dog samples described earlier, which did not show cross-reactivity with SARS-CoV-2 S1 in our ELISAs.

## Discussion

Because SARS-CoV-2 can infect cats and dogs, the virus might spread in this population and animals might act as a reservoir with the possibility of animal-to-human transmission. Although so far the pandemic has been driven by human-to-human transmission, it is useful to know whether domestic animals can play a role in maintenance and spread of SARS-CoV-2 infections, as underscored by the recent reports that workers from mink farms had acquired SARS-CoV-2 from minks (*24*,*25*). For these studies, verified serologic assays that detect virus-specific antibody responses in cats and dogs are needed. In our study, we modified assays used in human epidemiologic studies and validated ELISAs to detect SARS-CoV-2 S1 and RBD antibodies and VN by using pseudotyped SARS2-VSV for screening cat and dog samples. We defined seropositivity on the basis of results for positive samples from the SARS-CoV-2–exposed cohort.

We also showed that N protein, which is used in serologic studies with human samples (*19*,*26*), lacks discriminating power. We found a poor correlation between the results of the N protein ELISA and the VNT and the S1 and RBD ELISAs. Several of the pre–COVID-19 samples were positive in the N protein ELISA, probably because of antigenic cross-reactivity between SARS-CoV-2 and FCoV type I N proteins. These data validate SARS-CoV-2 S1 and RBD and exclude N protein as antigens for serologic screening of cat and dog serum samples. A similar phenomenon was also reported between porcine epidemic diarrhea virus and porcine transmissible gastroenteritis virus (*27*). Therefore, N protein cannot be used for serologic screening of samples from cats and dogs.

To date, most studies focused on molecular detection of SARS-CoV-2 in exposed animals, and virus detection is also used as the case definition by the World Organisation for Animal Health (*28*). However, serologic studies are needed to gain insights into the role of domestic animals in the epidemiology of the disease because they serve as a strong functional complement of molecular detection. In a recent molecular survey, no positive samples were detected for 4,000 samples from companion animals (cats, dogs, and horses) (*29*). However, serologic screening was not performed. In our study of samples from domestic animals with unknown SARS-CoV-2 exposure, we determined seroprevalences for SARS-CoV-2 of 0.4% for cats and 0.2% for dogs, which is lower than the prevalence rate of endemic coronaviruses, such as FCoV and CCoV, and also lower than the seroprevalence estimate in human populations in the Netherlands (2.7%–9.5%) at the period of sample collection (*30*,*31*). In our study, we also found a much lower seroprevalence than for domestic cats and dogs in northern Italy, where >3% of samples were seropositive (*32*). However, all of these animals lived in SARS-CoV-2–positive households or in severely affected geographic areas. Such observations demonstrate that cats and dogs can acquire SARS-CoV-2 infection, but that the virus was not widely circulating in the cat and dog populations of the Netherlands at the time of sampling (April–May 2020).

VN assays are considered to be the reference standard for assessing immunity to many coronavirus infections based on their exceptional specificity (*33*). Therefore, we defined a sample positive when the S1 and RBD ELISA results were positive and confirmed by VN. In our screening, 4 cat samples were positive for S1 and RBD by ELISAs, but failed to neutralize SARS2-VSV infection and were defined as suspected. This finding might be related to individual differences in development of neutralizing antibodies, such as different levels of SARS-CoV-2 exposure and time of sampling postinfection. In humans with asymptomatic or mild infection of Middle East respiratory syndrome coronavirus and SARS-CoV-2, samples were seropositive but failed to neutralize virus infection (*33*,*34*). Moreover, 14 samples reacted only with S1 or RBD in ELISAs and were defined as seronegative because they did not reach our diagnostic threshold (Table 2).

One limitation of our study is that lack of knowledge on the kinetics of SARS-CoV-2 antibodies in cats and dogs limits the setup of validated serologic assays. VN assays are considered to be a standard, but little is known regarding sensitivity compared with S1 or RBD ELISAs for identifying SARS-CoV-2 infections. Future studies require systematic analyses of development of antibody responses against different antigens in cats and dogs experimentally infected with SARS-CoV-2. In addition, regarding sampling methods used for the 2020 cohort, it is not possible to trace the health status and the level of SARS-CoV-2 exposure for those animals. Therefore, we cannot make any associations between antibody levels and clinical status. Also, our data report mainly SARS-CoV-2 seroprevalence during the first wave of the COVID-19 pandemic (April–May 2020). Whether seroprevalence is different during the second wave of the pandemic remains unknown. Moreover, possible implication of the emergence of new SARS-CoV-2 variant strains on the infection of animals remains to be established.

Overall, we developed and validated a set of serologic assays, and conducted seroprevalence study of SARS-CoV-2 infection in domestic cats and dogs in the Netherlands. The general prevalence rate was low at the time of sampling, indicating that cats and dogs are probably incidental hosts because of occasional SARS-CoV-2 spillover from humans. However, continued serosurveillance is needed to monitor possible, sustained transmission of SARS-CoV-2 infection in companion animals and a wider range of other animal species. This need is especially required because the incidence of COVID-19 in humans is still increasing in several parts of the world.
